# Automated image analysis detects aging in clinical-grade mesenchymal stromal cell cultures

**DOI:** 10.1186/s13287-017-0740-x

**Published:** 2018-01-10

**Authors:** S. Oja, P. Komulainen, A. Penttilä, J. Nystedt, M. Korhonen

**Affiliations:** 1 Advanced Cell Therapy Centre, Finnish Red Cross Blood Service, Kivihaantie 7, FI-00310 Helsinki, Finland; 2 Institute of Biomedicine, Department of Anatomy, University of Helsinki, Haartmaninkatu 8, FI-00290 Helsinki, Finland; 3 Department of Physics, University of Helsinki, P.O. Box 64, FI-00014 Helsinki, Finland; 4 Division of Hemato-Oncology and Stem Cell Transplantation, Hospital for Children and Adolescents, Helsinki University Central Hospital, FI-00290 Helsinki, Finland

**Keywords:** Mesenchymal stromal cells, MSC, Aging, Senescence, Quality control, Morphology, Imaging, Cell manufacturing, Cell therapy

## Abstract

**Background:**

Senescent cells are undesirable in cell therapy products due to reduced therapeutic activity and risk of aberrant cellular effects, and methods for assessing senescence are needed. Early-passage mesenchymal stromal cells (MSCs) are known to be small and spindle-shaped but become enlarged upon cell aging. Indeed, cell morphology is routinely evaluated during MSC production using subjective methods. We have therefore explored the possibility of utilizing automated imaging-based analysis of cell morphology in clinical cell manufacturing.

**Methods:**

An imaging system was adopted for analyzing changes in cell morphology of bone marrow-derived MSCs during long-term culture. Cells taken from the cultures at the desired passages were plated at low density for imaging, representing morphological changes observed in the clinical-grade cultures. The manifestations of aging and onset of senescence were monitored by population doubling numbers, expression of p16^INK4a^ and p21^Cip1/Waf1^, β-galactosidase activity, and telomeric terminal restriction fragment analysis.

**Results:**

Cell area was the most statistically significant and practical parameter for describing morphological changes, correlating with biochemical senescence markers. MSCs from passages 1 (p1) and 3 (p3) were remarkably uniform in size, with cell areas between 1800 and 2500 μm^2^. At p5 the cells began to enlarge resulting in a 4.8-fold increase at p6–9 as compared to p1. The expression of p16^INK4a^ and activity of β-galactosidase had a strong correlation with the increase in cell area, whereas the expression of p21^Cip1/Waf1^ reached its maximum at the onset of growth arrest and subsequently decreased. Mean telomere length shortened at an apparently constant rate during culture, from 8.2 ± 0.3 kbp at p1, reaching 6.08 ± 0.6 kbp at senescence.

**Conclusions:**

Imaging analysis of cell morphology is a useful tool for evaluating aging in cell cultures throughout the lifespan of MSCs. Our findings suggest that imaging analysis can reproducibly detect aging-related changes in cell morphology in MSC cultures. These findings suggest that cell morphology is still a supreme measure of cell quality and may be utilized to develop new noninvasive imaging-based methods to screen and quantitate aging in clinical-grade cell cultures.

**Electronic supplementary material:**

The online version of this article (10.1186/s13287-017-0740-x) contains supplementary material, which is available to authorized users.

## Background

Mesenchymal stromal cells (MSCs) are intensively studied for use in advanced cell therapies. Because of their strong immunosuppressive potential, MSCs have been used to treat acute and chronic graft-versus-host disease (GVHD) [1] and may prove beneficial for Crohn’s disease or other immunological disorders [2]. In addition to their immunomodulatory properties, MSCs can differentiate into osteogenic, adipogenic, and chondrogenic lineages [3], and therefore could be utilized in various tissue regeneration applications [2].

MSCs are a small population of multipotent stromal cells which can be isolated from various sources such as bone marrow, adipose tissue, or umbilical cord blood [4–7]. MSC cultures are heterogeneous, and only a subpopulation of the cells are self-renewing and may be considered multipotent stem cells [8]. Clinical-grade MSCs are commonly isolated from bone marrow aspirates, where they comprise 0.001–0.01% of the mononuclear cell fraction [9]. Because MSC therapies for GVHD require a large number of cells, MSCs need to be expanded extensively in vitro during production [2, 10].

Contrary to pluripotent stem cells, MSCs are somatic cells with a limited lifespan [11, 12]. Aging of cultured MSCs can be considered to begin when the primary culture has been established. Gradual aging leads finally to senescence, where cell division is permanently arrested, but the cells remain alive and metabolically active until cell death [13, 14]. Replicative senescence is induced through exhaustion of division potential, but senescence may also be initiated by DNA damage or stressors such as suboptimal culturing conditions [13, 15].

Telomeres shorten with each round of cell division and reflect the biological age of cells and the organism [16]. Senescence is triggered when a cell’s shortest telomere has reached its critical minimum length [17, 18]. In addition to telomere shortening, aging and senescence are also accompanied by exhaustion of cell division [11], accumulation of senescence-associated β-galactosidase (SA-β-gal) [19], and an increase in expression of the cyclin-dependent kinase inhibitor p16^INK4a^ [20, 21], which is responsible for permanent cell cycle arrest together with the p53/p21^Cip1/Waf1^ pathway [22]. However, the most readily observed indicators of cellular senescence are changes in cell size and morphology from small and spindle-shaped into large and irregular cell shapes [12, 23].

Aging affects the functionality of cultured MSCs, ultimately leading to reduced differentiation [23] and immunosuppressive potential [24, 25], which are the primary therapeutic mechanisms of MSCs in cellular therapies. Senescence increases the risk of genetic instability and cell transformation [26] but, importantly, senescent cells affect their microenvironment by secreting cytokines, chemokines, and proteases, known as the senescence-associated secretory phenotype (SASP) [27, 28]. Cells with SASP have diverse effects including the ability to promote tumorigenesis and facilitate proliferation and invasiveness of malignant cells [27].

Cells manufactured for therapeutic use must fulfill high quality criteria. The International Society for Cellular Therapy (ISCT) minimal criteria for MSCs define them as adherent fibroblastoid cells which express characteristic surface antigens, lack hematopoietic markers, and are able to differentiate into osteoblasts, adipocytes, and chondroblasts [29]. For clinical-grade MSCs, quality criteria also require sterility and normal karyotype [30].

Researchers and regulators agree that cell therapy products should be evaluated for senescence because it may compromise cell quality and functionality [26]. Senescence and transformation are tightly connected, and transformation is considered a serious safety risk of cell therapies. However, it seems unlikely that genetic instability of human MSCs will lead to transformation [31–33], but rather the altered functions of the senescent cells are a more likely risk to product quality and safety [28]. Currently, there are a variety of methods available for senescence assessment, but specific markers are still lacking. Routine MSC manufacturing would benefit from a robust and simple method for screening senescent cells from cultures.

We have shown that MSCs of consistent quality can be produced for clinical research programs [1, 10]. In this study, we have set up an analysis method based on automated image analysis for quantification of age-related changes in MSC morphology. We analyzed whether changes in morphology could be quantified using an image analysis system, and how these changes accompany cell aging. Our aim was to establish and test the applicability of automated image analysis to clinical-grade cell production as a screening method for cellular senescence.

## Methods

### Bone marrow MSC cultures

MSCs were obtained from bone marrow aspirates taken from the iliac crests of six healthy adult volunteer donors, aged 20–40 years, after written informed consent. Donors were coded for anonymity (MSC-1 to MSC-6). All donor protocols were approved by the Ethical Committee of the Hospital District of Helsinki and Uusimaa, Finland.

MSCs were isolated from bone marrow aspirates and cultured continuously from passage p0 to senescence. For this purpose, the cell culture was considered senescent if the confluency remained less than 30% after 2 weeks of culturing and cell appearance was typical of senescence, i.e., large and flat cell morphology, irregular cell shapes and granularity. MSCs were cultured in basic medium consisting of Dulbecco’s modified Eagle’s medium (DMEM) low glucose supplemented with 100 U/ml penicillin, 100 μg/ml streptomycin (all from Life Technologies, Thermo Fischer Scientific, Waltham, MA, USA), 40 IU/ml heparin (Heparin LEO 5000 IE/KY/ml, Leo Pharma, Malmö, Sweden), and 10% platelet lysate (Finnish Red Cross Blood Service, named PL1 in Laitinen et al. 2015 [10]). Complete protocols for cell isolation and culturing procedures are described elsewhere [10].

Continuous cultures were performed both on a small, research laboratory scale (MSC-1 to MSC-3) and also using large culture chambers (MSC-4 to MSC-6). For small-scale cultures, 75 cm^2^ and 175 cm^2^ cell culture flasks (Corning, NY, USA) were used, the 175 cm^2^ flask being used at p1, p3, p5, p7, and p9 in order to obtain sufficient cells for analyses. Large scale cultures were performed using Cell Stack® (Corning) two-layer cell culture chambers with a growth area of 1272 cm^2^ to mimic culturing vessels used in cell manufacturing. Cells were collected for analyses at p1, p3, p5, and pSEN, the latter representing the final collected passages (p6–9) where cells were classified senescent as defined earlier.

Confluency and culture characteristics were monitored during cell culturing by a Nikon Eclipse TS-100 phase contrast and inverted microscope with 4× magnification (Nikon, Japan). Upon reaching 80% confluency, the cells were harvested using TrypLE Select CTS (Invitrogen, MA, USA). Cell count and viability were measured using NucleoCounter (Chemometec, Denmark) and cells were reseeded at a density of 1000 cells/cm^2^ and passaged continuously until senescent.

Population doubling (PD) was calculated for each passage according to PD = log_2_ (*N*_H_/*N*_1_), where *N*_1_ is the number of seeded cells/cm^2^ (starting count), and *N*_H_ is the number of harvested cells/cm^2^. The CFU-F count of the original bone marrow aspirate at passage 0 was used as the first *N*_1_ [10]. The successive PDs obtained at each passage were added together to obtain the cumulative PD number for a continuous culture.

### Characterization of the MSCs

Characterization of the MSCs was performed according to ISCT guidelines [29]. Flow cytometric analysis of the cell surface antigens CD44, CD49e, CD13, CD90, CD73, CD29, CD105, CD14, CD19, CD34, CD45, HLA-ABC, and HLA-DR was performed using the Navios Cytometer (Beckmann Coulter, IN, USA) and analyzed using FlowJo software (version 7.4.1, Tree Star Inc., CA, USA). At least 5000 cells were analyzed per sample. All antibodies were purchased from BD Biosciences (CA, USA), except HLA-DR IgG1 isotype control, which was from Abcam (UK).

For osteogenic and adipogenic differentiation assays MSCs were plated at a density of 1000 cells/cm^2^ in six-well plates and cultured to confluency in basic medium. Osteogenic and adipogenic differentiation assays were performed according to Laitinen et al. [10]. Osteogenic differentiation was detected by von Kossa staining [34–38], and adipogenic differentiation by Sudan III staining [36–38].

### Sample preparation for the image analysis

The cells from passages 1, 3, 5, and SEN (passages 6–9) were plated at 3000 cells/cm^2^ on six-well plates for subsequent fixation and staining. After attachment and spreading for 48 h, the cells were fixed with 4% paraformaldehyde (Sigma, MO, USA) and stored at 4 °C in 0.02% sodium azide/phosphate-buffered saline (PBS). Fixed cells were permeabilized with 0.1% Triton X-100/PBS (Sigma), and the cytoplasm was stained using Cell Mask Deep Red Stain (1 μg/ml, Life Technologies) and the nuclei with DAPI (0.125 μg/ml, Sigma).

### Image calibration and acquisition settings

**Fig. 1:**
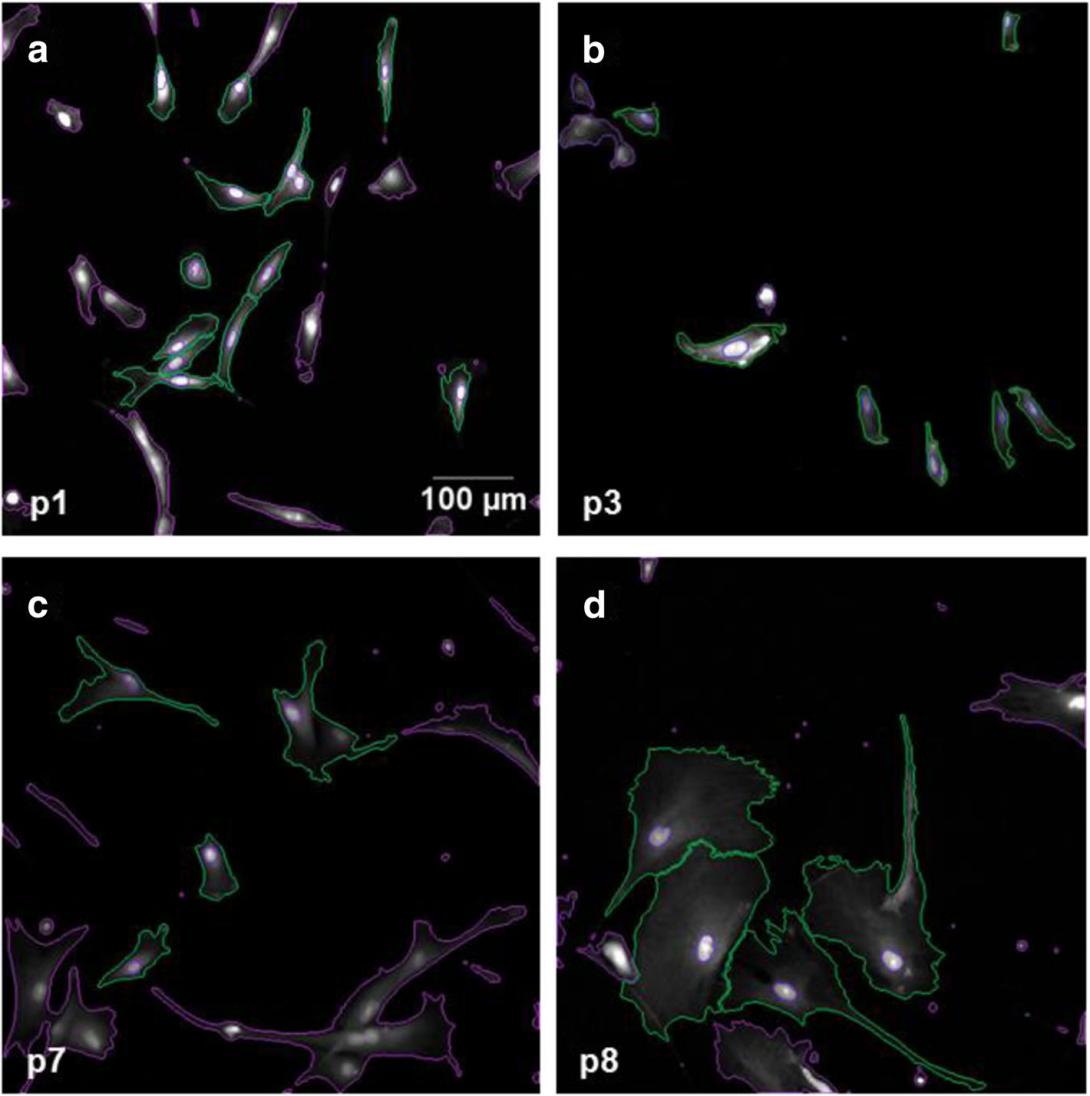
Automated Cell Insight analysis (representative figures). The images were acquired from passages p1, 3, 5, and pSEN (**a**–**d**) from six donors using 10× magnification. The cell nuclei and the cytoplasm were stained with DAPI and Cell Mask Deep Red Stain, respectively. Cells accepted for the analysis are lined in *green* and excluded cells in *magenta*

For calibration purposes, a range of several test MSCs of different passages was plated, fixed, and imaged as described in the previous paragraph. Calibration data were analyzed several times from each sample, and gating parameters were adjusted manually after each iteration. These parameters were the minimum object size, the threshold signal intensity when two objects are considered separate, and the threshold for finding an object’s edge. An object was considered a cell when it included only one nucleus and it was wholly contained within the image field (Fig. 1a-d). The program was also instructed to accept only objects that were larger than a set size limit, 765 μm^2^. This resulted in a final imaging protocol yielding only a few false positives, i.e., low proportion of cell culture debris being classified as viable cells or two cells counted as one, and a moderate level of false negatives (viable cells gated out). We found that it was necessary to adjust the exposure time separately for each run because of dye bleaching. Adjustment was performed automatically using the first field of each run. The image was automatically refocused after every ten fields, resulting in reasonable image quality and running time.

### Image acquisition

The images were acquired using a high-content screening microscope (Cell Insight, Thermo Scientific, IL, USA) with a 10× objective (Olympus, Japan). Signals from Cell Mask and DAPI stains were acquired on separate channels using filters at 630 nm and 386 nm, respectively. Three to six wells of a six-well plate were imaged for each analysis resulting in 999–1998 images per channel at each run. The images were taken starting from the well center in a spiral fashion to minimize the optical distortion caused by the convexity of the well bottom near the edges.

### Image analysis

The resulting images were analyzed using Cell Omics Morphology Explorer software (version V4, Thermo Scientific). Altogether nine morphological parameters were measured for every cell; four for size (length, width, area, and perimeter) and five for shape (perimeter to area ratio, length to width ratio, boxed frame ratio, convex hull area ratio, convex hull perimeter ratio) (Table 1). Boxed frame ratio refers to the cell area, which is defined by fitting a cell inside a rectangular box after which the ratio of the cell area to the bounding box area was calculated. Convex hull was defined by stretching an imaginary rubber band around the cell and calculating the area demarcated by the rubber band. Convex hull area ratio was determined by calculating the ratio of rubber band framing area to the cell area calculated according to cytoplasmic stain.

**Table 1:** Parameters with explanations acquired using automated image analysis

Size	
Length	Length of image-aligned bounding box of cell
Width	Width of image-aligned bounding box of cell
Area	Area of the cell
Perimeter	Perimeter of the cell
Shape	
Perimeter to area ratio	The ratio of the cell perimeter to 4π area of the cell. A measure of the roundness of the cell.
Length to width ratio	The ratio of the length and width of the bounding box of the cell.
Boxed frame ratio	The ratio of the cell area to the bounding box area.
Convex hull area ratio	Ratio of convex hull (“rubber band bounding the cell”) area to the cell area. Convex hull area ratio is greater in cells with long pointy processes compared to cells with no sharply shaped processes.
Convex hull perimeter ratio	The ratio of convex hull length to the detected cell edge length.

Altogether nine parameters describing cell morphology were measured using the Cell Insight platform

### Telomere length analysis

Mean telomere lengths were measured by Southern blot analysis of terminal restriction fragments (TRFs) [39]. Genomic DNA from snap-frozen cell pellets was purified using the Qiagen DNeasy Blood and Tissue Kit (Qiagen, MD, USA) and precipitated with sodium acetate and ethanol. The integrity of the purified DNA was evaluated by electrophoresis on a 1% agarose gel. Telomere length analysis was performed from triplicate samples using a TeloTAGGG Telomere Length Assay Kit (Roche, Switzerland). Extracted genomic DNA (2 μg) was digested using *RsaI* and *HinfI* enzymes and electrophoresed on a 0.8% agarose gel 125 V (4 V/cm) for 4.5 h. The separated DNA was transferred to a positively charged nylon membrane (Roche, Switzerland) by Southern blotting using 20× saline-sodium citrate buffer (SSC), after which the transferred DNA was UV-crosslinked at 120 μJ/cm^2^ (UVP CL-100, UK). The blot was hybridized overnight with a digoxigenin (DIG)-labeled telomere-specific probe (TTAGGG), which was subsequently detected using an alkaline phosphatase-labeled anti-DIG antibody and CDP-Star chemiluminescent substrate and used to expose an autoradiography film (GE Healthcare, WI, USA). The average length (kilobase pairs, kbp) of the telomeric TRFs were calculated using ImageJ analysis software [40] and Excel software (Microsoft, WA, USA) according to mean TRF = Σ (OD_i_ × L_i_)/Σ (OD_i_) where OD_i_ is optical density and L_i_ is the length of the TRF at position i. TRF signals between 3 and 20 kbp were used for telomere length measurements [39].

### Immunoblotting of the cell cycle regulatory proteins

Snap-frozen cell pellets were lysed in RIPA buffer (Thermo Scientific) containing 1% (v/v) Protease Inhibitor Cocktail (Sigma). Protein concentrations were determined using the BCA Protein Assay Kit (Pierce, IL, USA); 20 μg of total protein was run on a 12% TGX gel (Bio-Rad, CA, USA) and electrotransferred to a Hybond ECL nitrocellulose membrane (GE Healthcare). The membrane was blocked with 5% milk in TBST and immunoblotted using anti-p16^INK4a^ (1:500; clone G175-1239) and anti-p21^Cip1/Waf1^ (1:250; Clone SXM30) (both BD Pharmingen, CA, USA) primary antibodies. β-Actin (1:8000; monoclonal anti-β-actin, clone AC-74; Sigma) was used as a loading control. Horseradish peroxidase (HRP)-conjugated polyclonal anti-mouse immunoglobulin was used as the secondary antibody (1:1000; Dako Cytomation, Denmark). The signal was detected using a chemiluminescent detection system (ECL; GE Healthcare), and the band intensities were quantified using a Scanjet G4050 scanner (Hewlett-Packard, CA, USA) and Image J analysis software [40].

### Senescence-associated β-galactosidase assay

SA-β-gal activity was measured using the Cellular Senescence Assay Kit (Cell Biolabs, CA, USA) according to the manufacturer’s instructions. Cells for the assay were cultured until 80% confluency and samples were collected. Samples were lysed, and equal amounts of total protein were loaded to the assay. Fluorescent signals were read using a ClarioStar monochromator plate reader (BMG Labtech, Germany) with excitation at 360 nm and emission at 465 nm.

### Statistical analysis

The statistical analysis of the data was performed using Mathematica software (version 11.0.1, Wolfram Research, IL, USA). The imaging data were cleaned by removing outliers and by applying the Box-Cox transformation. The outlier removal was performed by trimming out a portion of the smallest and largest values of the corresponding variable. The Box-Cox transformation for a variable *y* with a parameter *λ* is of the form *y’* = (*yλ*-1)/*λ* if *λ* ≠ 0, and *y’* = log(*y’*) if *λ* = 0. Analysis of variance (ANOVA) tests were used, one- and two-way, for analyzing the group differences. If differences were statistically significant with more than 95% confidence, paired differences between the groups were tested with Bonferroni-corrected Student’s *t* tests. The two-sample Kolmogorov-Smirnov distribution test was used to test the hypothesis of two distributions being the same for the data in Fig. 3.

Correlation analyses between mean cell area measurements after outlier removal and aging-related markers were performed by determining Pearson correlation coefficients. Visualization of the correlations was done by a heat map and principal component analysis using R language for statistical programming and graphical analysis.

## Results

### Characterization of MSCs

MSCs were characterized by immunophenotype, adherence to plastic, and by the ability to differentiate into osteogenic and adipogenic lineages. Samples from all donors expressed the surface antigens CD13, CD44, CD49e, CD90, CD73, CD29, CD105, and HLA-ABC, and were negative for CD14, CD19, CD34, and CD45 (Additional file 1: Table S1). In distinction from ISCT guidelines [29], the cells from all donors expressed HLA-DR (average 27.1% positive cells, range 7.5–47.4%) as we have reported previously for cells grown in platelet lysate [10]. All cells differentiated into osteogenic and adipogenic lineages (Additional file 2: Figure S1 and Additional file 3: Figure S2).

### Growth kinetics

**Fig. 2:**
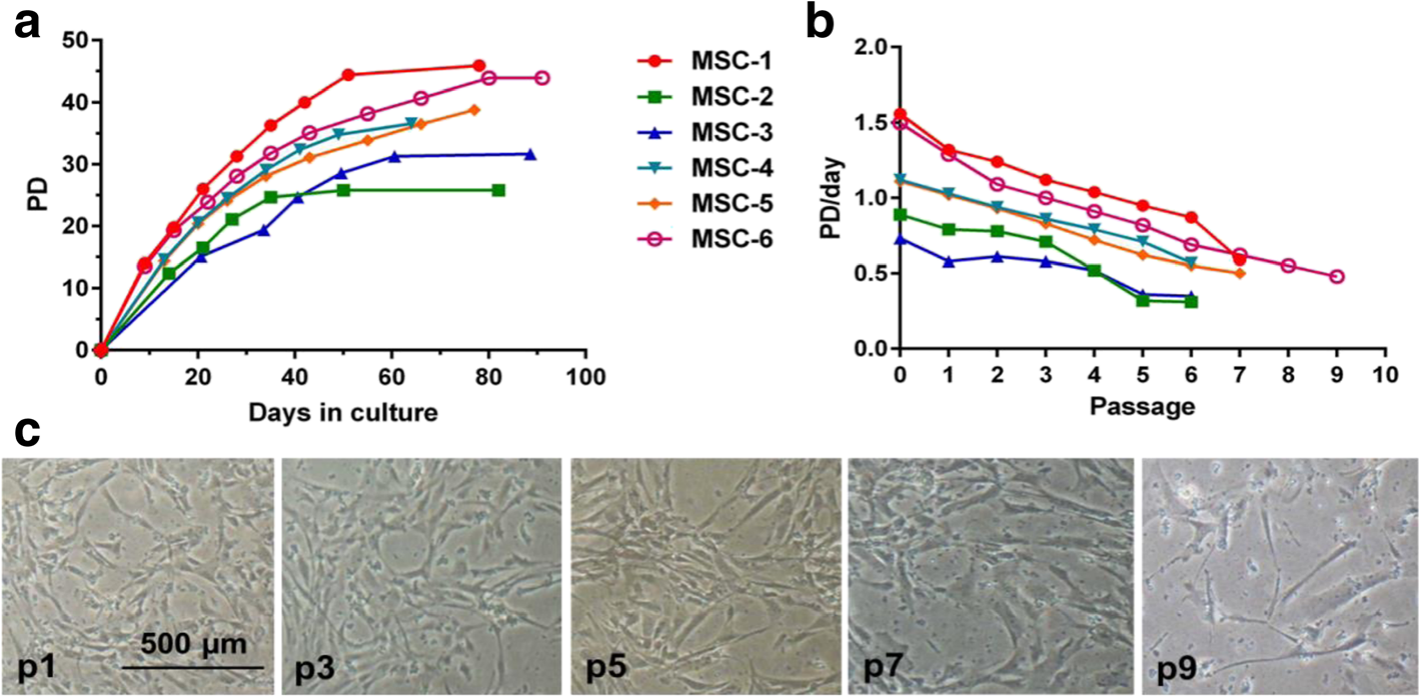
Growth kinetics from long-term continuous cultures of MSCs. Mesenchymal stromal cells (*MSCs*) were isolated from bone marrow aspirates collected from healthy volunteer donors (*n* = 6; MSC-1 to MSC-6) and cultured continuously from primary cultures (passage 0) to senescence. **a** Cumulative population doubling numbers (*PD*) are presented as a function of culturing time. Each point in the growth curve indicates an individual cell passaging event starting from passage 0. **b** The rate of cell division is presented as PD/culturing day per passage (*n* = 6). **c** Representative morphology and culture characteristics of long-term MSC cultures from passages 1, 3, 5, 7, and 9 (donor MSC-6) imaged using 4× magnification. The respective passage (*p*) numbers are shown in each micrograph

Average culturing time for MSC-1 to MSC-6 from primary cultures to senescence was 80 ± 10 days (Fig. 2a). Cells from almost all donors were in logarithmic growth phase (average 0.85 PD/day) until passages 5–6, after which the rate of cell population doubling decreased to 0.31–0.82 doublings/day (Fig. 2b). The cultures ceased proliferation at passages p5–p9 after 38.5 ± 5.6 PDs. Division of donor MSC-2 cells was arrested early, after 25 PDs at passage 5. Also, donor MSC-3 cells showed earlier exhaustion of division potential. MSCs from the first passages (p1–3) were small and spindle-shaped, whereas cells from passage 5 manifested changes in morphology (Fig. 2c). The pSEN passages (the final collected passages, p6–9) showed typical characteristics of senescent cells such as large size, irregular shapes, and granularity (Fig. 2c).

### Statistical analysis of cell morphology

Image analysis of cells produced morphology for a total of 313,141 cells. It is evident that this large dataset contains cases that the automated image analysis system has misinterpreted, e.g., classifying overlapping cells as one or debris as whole cells. Therefore, the data were trimmed before the actual analysis to remove artificial outlier values. We selected to trim out 5% of the smallest and largest values. As a consequence, the total number of cells for the analysis was 281,827.

Even after the outlier removal, the morphological variable distributions were remarkably non-normal. Almost all the distributions were highly skewed to the right, except the boxed frame ratio and the convex hull perimeter ratio, which were skewed to the left. For example, the average skewness value for the cell area was 0.96 over the donor/passage groups. Therefore, the Box-Cox transformation with *λ* = 0 (log-transform) was applied to all the variables, except for the boxed frame ratio and the convex hull perimeter ratio variables, for which *λ* = 2 transform was performed. After the transform, the variable distributions were closer to normal, and the average skewness for the transformed cell area, for example, was 0.091.

In order to select the best morphological variable associated with the PD number, we fitted linear first-degree models with each morphological variable as the explanatory variable for the PD. Furthermore, we tested both the donor/passage subgroup mean value and the standard deviation (SD) value as the explanatory variable. The *R*^2^ coefficient of determination for the models was highest for the variables describing the size of the cell, i.e., area, perimeter, length, and width. Also, the SD of the group gave higher *R*^2^ values than the mean of the group. For the SD of the size variables, the *R*^2^ values were 48% for the area, 47% for length, 43% for perimeter, and 38% for width. Thus, we conclude that the cell area or the uniformness of the cell area is the best indicator of the increasing PD numbers.

To statistically verify that there are significant differences in cell area between the passages, we analyzed the donor/passage groups using a two-way ANOVA test. For the cell area, the result showed that donors, passages, and donor/passage groups all had non-constant cell area values with *p* values practically zero. Furthermore, the pairwise post-tests showed that all the donor-donor or passage-passage differences are statistically significant.

### Extended in vitro culture is associated with an increase in cell size

**Fig. 3:**
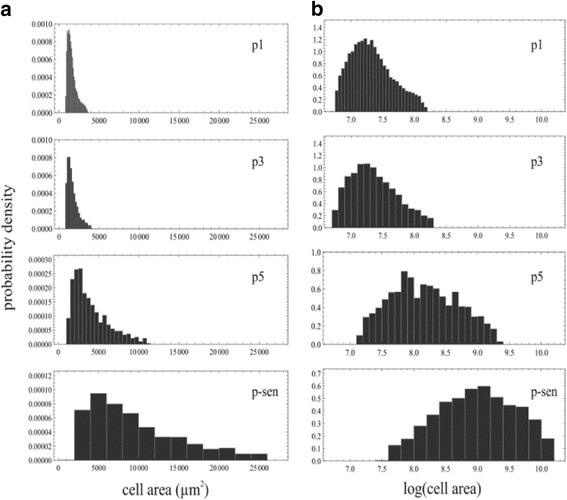
Mean cell area variability in MSCs. Representative graphs of the size distributions of MSCs according to **a** cell area and **b** log cell area, acquired from filtered data. Maximum value in *y*-axis (probability) for passage p5 and senescent passage pSEN graphs was set lower than in passage p1 and 3 graphs for better visualization of the size distribution. The two-sample Kolmogorov-Smirnov test was used to verify the similarity between the distributions for passage 1 against the passages 3, 5, and SEN. The *p* values were all well below any sensible rejection limits, starting from 1.4 × 10^–5^ for the passage 3 distributions. Thus, we can safely reject the hypothesis that the distributions between the passages would be the same. The non-parametric Kolmogorov-Smirnov test will give the same *p* values for the original (**a**) and the log-transformed (**b**) distributions

**Fig. 4:**
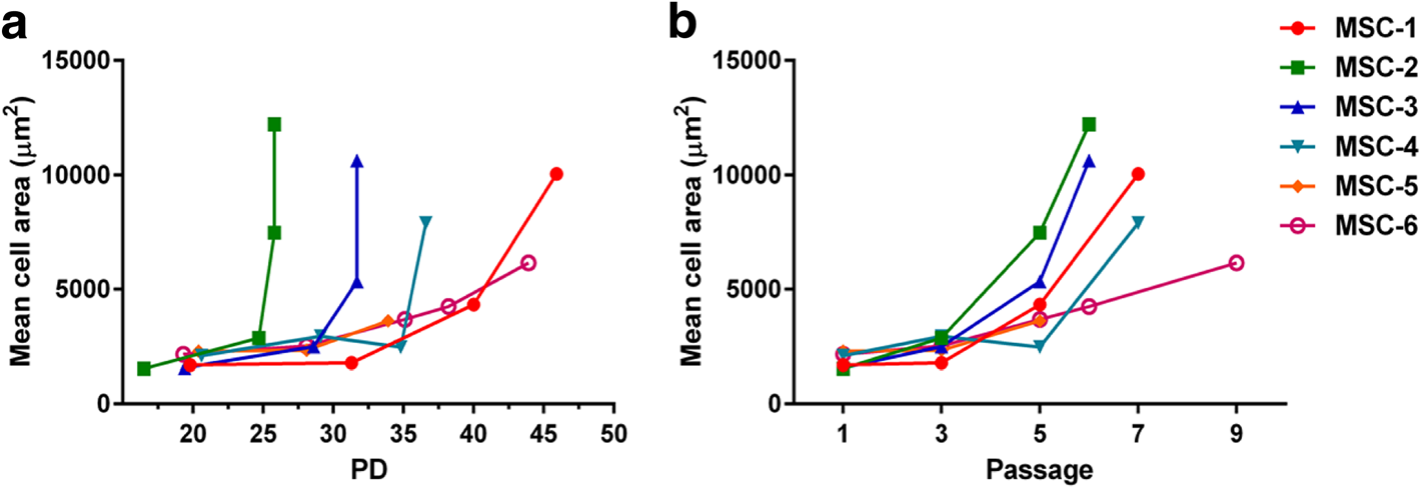
Mean cell area increases with extended in vitro cell culture. Mean cell area is presented in function of **a** population doubling (*PD*) numbers and **b** passages after outlier filtering (*n* = 6). Each point in the graph presents imaged passages 1, 3, 5 or p6–9 (pSEN). Standard deviations are omitted from the graph for clarity

We then systematically analyzed cell area and its distribution in continuous MSC cultures. Passage 1 MSCs were a homogenous population of small cells (Fig. 3a). During further culture, the distribution of cell sizes was broadened towards the right, slightly at passage 3 and markedly at passage 5 and onwards (Fig. 3a and b). Nevertheless, a population of small cells could be detected even at pSEN passages (Fig. 3a). The mean cell area at passage 1 (19.3 ± 1.5 PD) was 1827 ± 329 μm^2^ (Fig. 4a and b, Table 2). A moderate increase in cell area was seen at passage 3 (28.3 ± 2.1 PD) with an average of 2352 ± 386 μm^2^, and further at passage p5 (33.5 PD) resulting in cell areas of 4198 ± 1628 μm^2^. The average cell size increased remarkably at pSEN when cell areas were 4.8-fold higher compared to passage 1 with cell areas of 8744 ± 2494 μm^2^ (Table 2). Maximum PD numbers at pSEN were 36.6 ± 7.1. The increase in cell size is particularly abrupt when plotted against PD numbers, reflecting the slowing of population growth at late passages (Fig. 4a). However, a marked escalation of cell gain upon senescence is also noted when cell size is plotted against passage number (Fig. 4b). Different cultures displayed the sharp escalation in cell size at different PDs, reflecting the variability at which the cultures reached senescence. The cells that were grown in small-scale culture flasks reached a larger size (10,368 ± 1013 μm^2^) at final passages than cells grown in large-scale vessels (6306 ± 1739 μm^2^) (Additional file 4: Figure S3). However, in both culture formats, a pronounced increase in cell area at final passages could be observed.

**Table 2:** Mean cell areas, corresponding population doubling numbers, and fold-changes in comparison to passage 1

Population doubling (passage)	Mean cell area (μm^2^)	Fold-change in cell area in comparison to passage 1
19.3 ± 1.5 (p1, *n* = 6)	1827 ± 329	
28.3 ± 2.1 (p3, *n* = 6)	2352 ± 386	1.3
33.5 ± 4.7 (p5, *n* = 6)	4198 ± 1628	2.3
36.6 ± 7.1 (pSEN, *n* = 5)	8744 ± 2494	4.8

Measurements from passages (p)1, 3, and 5 are performed from all six donors. Morphological analysis of passages 6–9 (pSEN) were acquired from *n* = 5 due to early senescence of donor MSC-2 cells

### Increase in cell size is accompanied by the expression of senescence markers

**Fig. 5:**
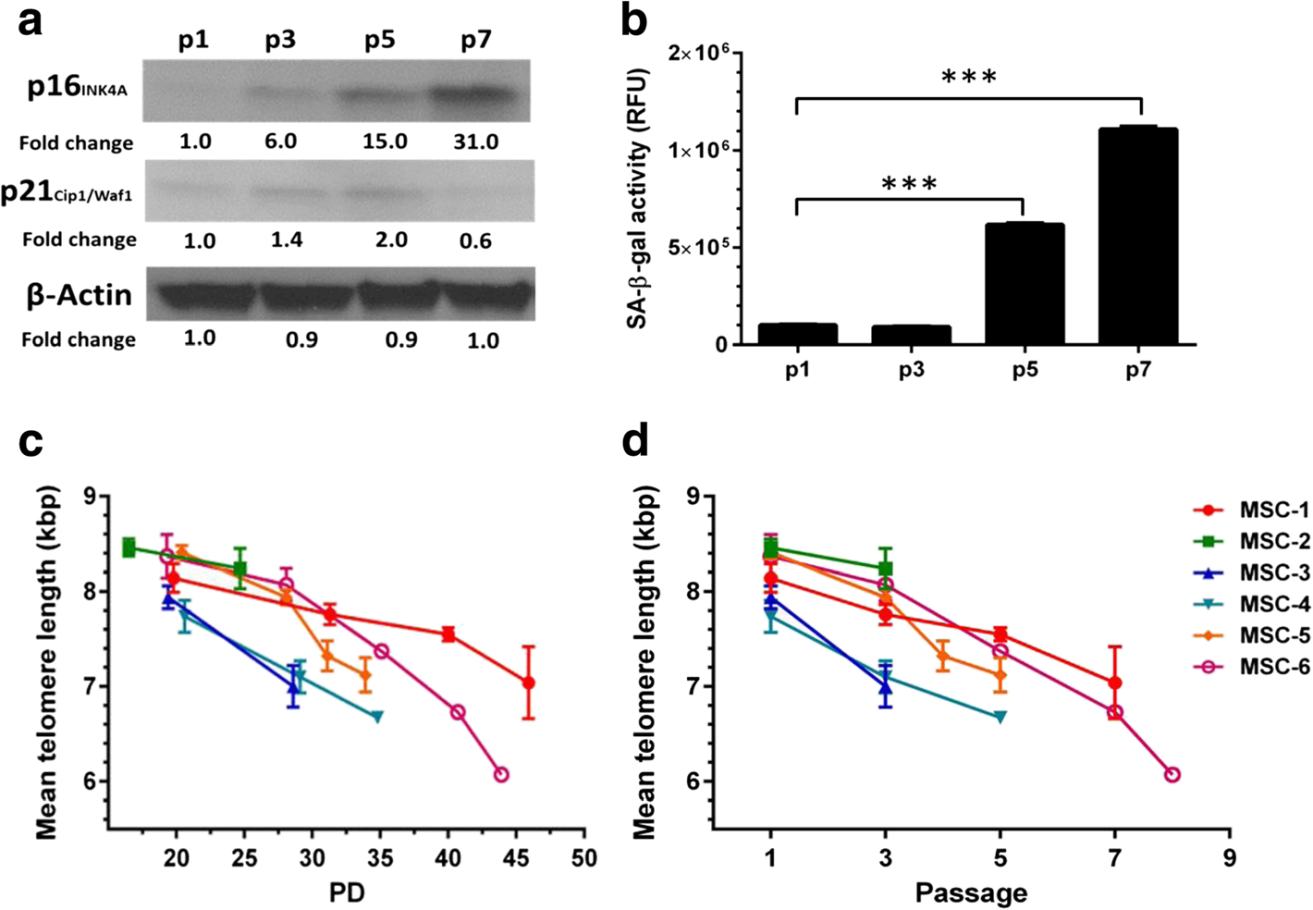
The expression of aging-related markers is increased at the onset of senescence. **a** A representative image of p16^INK4a^ and p21^Cip1/Waf1^ Western blot analyses (*n* = 6). Equal amounts of total protein (20 μg) were loaded onto the gel, and β-actin was used as a loading control. **b** Senescence-associated β-galactosidase (*SA-β-gal*) activity was measured from three donors using a quantitative activity assay. Equal amounts of total protein were used per sample, and all samples were analyzed in triplicate. The results are presented as relative fluorometric units (*RFU*). Bonferroni-corrected Student’s *t* test, ***p < 0.001. Mean telomere length was measured from six donors using terminal restriction fragment analysis (TRF) and presented as in function of **c** population doubling (*PD*) numbers and **d** passage. Mean telomere length is shown as kilobase pairs (kbp)

Cellular aging and senescence were characterized by Western blot analysis of the cyclin-dependent kinase inhibitors p16^INK4a^ and p21^Cip1/Waf1^, quantitative analysis of SA-β-gal activity, and telomere length analysis (Fig. 5). The expression of the cell cycle regulatory protein p16^INK4a^ at passages 1 and 3 was low but was markedly increased at passage 5 (33.5 ± 4.7 PD), average 19,5-fold compared to p1 (see Additional file 5: Figure S4). Maximal expression of p16^INK4a^ was seen at passages 6–7 (35.8 ± 5.9 to 40.5 ± 4.0 PD) (*n* = 5), with a 33-fold expression as compared to p1 (Fig. 5a; Additional file 5: Figure S4). Expression of p21^Cip1/Waf1^ remained moderate from passage 1 to pSEN, with a small increase seen after passage 3, but with diminished expression at passage 5 when p16^INK4a^ reached its maximum and cells entered senescence.

The mean telomere length was assessed using TRF analysis (Fig. 5c and d; Additional file 6: Figure S5). Telomeres shortened with a constant rate from passage 1 to pSEN for all donor-specific MSCs. Mean telomere length at passage 1 (19.3 ± 1.5 PD) was 8.2 ± 0.3 kbp (range 7.7–8.5 kbp) and 7.7 ± 0.5 kbp (range 7.0–8.2 kbp) at passage 3 (28.3 ± 2.1 PD). The mean telomere length at pSEN (36.6 ± 7.1 PD) was 6.8 ± 0.6 kbp (range 6.1–7.8 kbp). The average rate of telomere shortening was 36.1 ± 12.4 bp/PD (range 23.9–56.3 bp/PD).

**Fig. 6:**
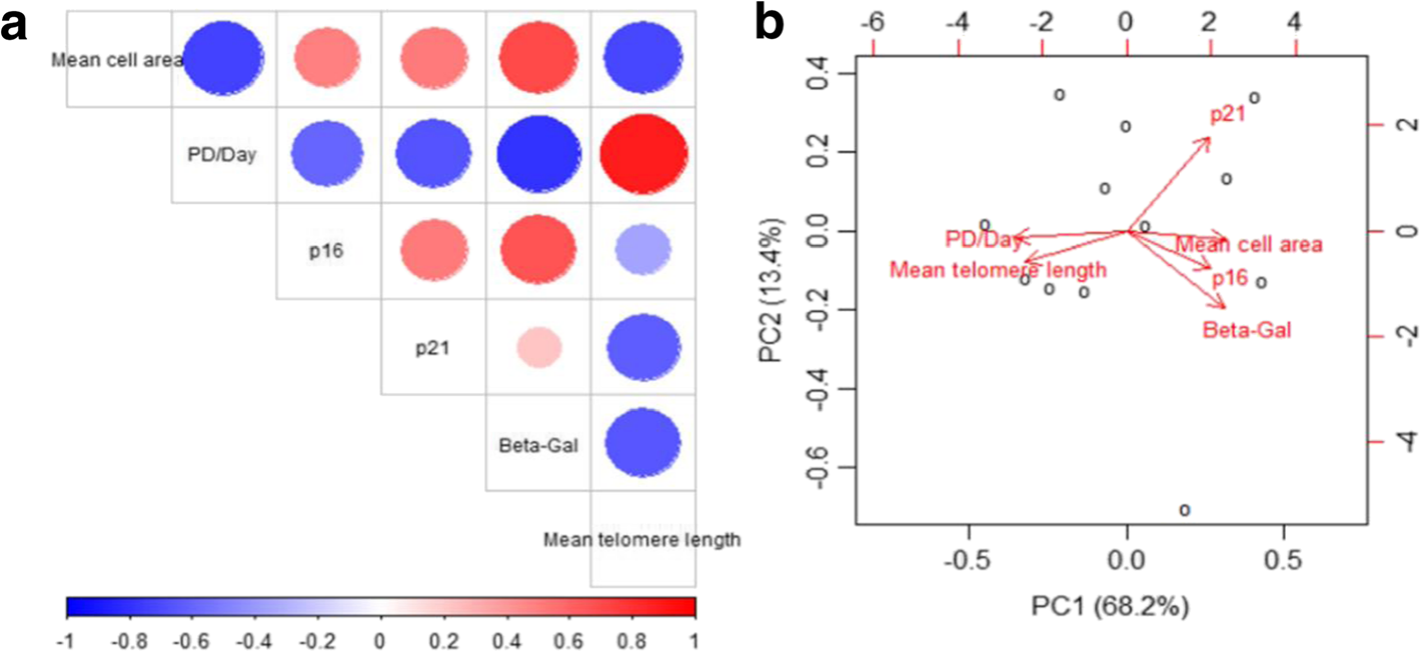
The expression of aging-related markers correlates with the increase in cell area. **a** Pearson correlation coefficients were determined to study the correlations between senescence markers and changes in cell size. Correlations are presented as a heat map, where *red circles* represent positive and *blue circles* negative correlations between senescence markers and cell area. The size of the circle indicates the value of the Pearson correlation coefficient. **b** Principal component analysis (PCA) was used to visualize the directions between the correlations in the mean cell area and the expression of aging-related markers. The PC1 axis aligns with mean cell area (positive) and population doublings (*PD*) per day (negative), and the mean telomere length, p16^INK4a^, and SA-β-gal variability also correlate with this axis. The PC2 axis is orthogonal to PC1, and mostly describes the variability in p21^Cip1/Waf1^ without a strong correlation to the mean cell area

Correlations between changes in the different aging-related parameters were analyzed by determining Pearson correlation coefficients between cell area and each aging-related marker (Table 3 and Fig. 6a). There were strong positive correlations (denoted by red circles in Fig. 6a) between cell area and p16^INK4a^ and between cell area and β-galactosidase, and strong negative correlations (shown as blue circles in Fig. 6a) between cell area and PD/day (pace of proliferation), and between cell area and shortening of mean telomere length. Changes in the expression of p21^Cip1/Waf1^ correlated weakly with the changes in cell size (Table 3 and Fig. 6a). Principal component (PC) analysis (Fig. 6b) visualized the correlations presented in Table 3 and Fig. 6a, showing strong interdependence between the expression of p16^INK4a^ and SA-β-gal and between PD/day and telomere length (oblique rotations). The expression of p21^Cip1/Waf1^ differed strongly along the PC2 axis from the other variables, indicating weak correlation with the increase in cell area (orthogonal rotation).

**Table 3:** Pearson correlation coefficients determined for mean cell area and each aging-related marker

	Mean cell area	PD/day	p16^INK4a^	p21^Cip1/Waf1^	SA-β-gal	Mean telomere length
Mean cell area	1.00	−0.74	0.50	0.52	0.70	-0.71
PD/day	−0.74	1.00	−0.59	−0.67	-0.80	0.89
p16^INK4a^	0.50	−0.59	1.00	0.52	0.66	-0.36
p21^Cip1/Waf1^	0.52	−0.67	0.52	1.00	0.23	-0.63
SA-β-gal	0.70	−0.80	0.66	0.23	1.00	-0.65
Mean telomere length	−0.71	0.89	−0.36	−0.63	-0.65	1.00

The data were collected from long-term cultures of three donors

*PD* population doubling, *SA-β-gal* senescence-associated β-galactosidase

## Discussion

Monitoring cell morphology is an essential part of clinical-grade MSC manufacturing [30]. However, simple methods for objective quantitation of changes in cell morphology during MSC culture are lacking. Aged and senescent cells should be avoided in clinical-grade cell cultures because they may compromise product quality and safety. Hence, methods for assessing the proportion of aged cells within MSC cultures are needed. In this study, we explored the use of an imaging analysis system for detecting aging-related changes in MSC cultures. We cultured MSCs from bone marrow aspirates of six healthy volunteer donors in animal serum-free culture conditions and measured the exact sizes of MSCs during long-term culture. We studied the correlations of morphological changes with senescence-associated parameters such as expression of the cyclin-dependent kinase inhibitors p16^INK4a^ and p21^Cip1/Waf1^, the activity of SA-β-gal, and shortening of mean telomere length.

The Cell Insight platform met our requirements for automated imaging analysis with readouts of several morphometric parameters. It is a high-content screening system for automated fluorescence image acquisition and quantitative analysis. As the ultimate goal was to test the applicability of the screening system in MSC manufacturing, we chose to use a conventional six-well culture plate format for imaging instead of, for example, small format glass-bottomed or coated plates that are considered optimal for imaging applications. This strategy might increase autofocus failures but, on the other hand, it better mimics culturing conditions for the MSCs. The six-well format also allowed us to avoid well edges in order to collect imaging data only from areas containing cells without distortions.

This study addresses three questions: 1) is it possible to quantify changes in cell morphology during long-term culture; 2) which is the most useful morphometric parameter for assessing cell aging; and 3) do the observed morphological changes correlate with the expression of senescence markers. Our aim was to test if aging-related morphological changes could be detected and quantified using an automated imaging analysis and to explore the use of such a method during the production of clinical-grade MSCs.

### Cells from the first passages are uniform in size

The major finding in this work was that cells from the first culture passages (p1 and p3) were remarkably uniform in size, correlating with low expression of senescence markers and a rapid logarithmic growth phase. The mean cell area and its variation began to increase rapidly after passage 5 (33.5 ± 4.7 PD), coincident with a significant increase in the expression of p16^INK4a^ and SA-β-gal (Fig. 5a; Additional file 5: Figure S4 and Additional file 6: Figure S5B). Expression of p21^Cip1/Waf1^ was moderately elevated during passages 3–5, before maximal expression of p16^INK4a^ was reached, and decreased when p16^INK4a^ expression attained its maximum (Fig. 5a; Additional file 5: Figure S4). Similar changes coincident with the onset of the senescence process and have also been reported by others [22].

The cell cultures of the study were performed both in small-scale culture flasks as well as in large two-layer cell stacks (1272 cm^2^), the latter mimicking the culture vessels used in clinical-grade manufacturing. Although the final cell area attained by the cells in the small-scale was larger than in large-scale cultures (Additional file 4: Figure S3), the phenomenon of abrupt area increase upon senescence was identical in both culture formats. No differences in senescence marker expression were observed either, suggesting that cell area monitoring can be applied to many types of cell culture systems.

We observed a conspicuously early increase in mean cell area with donor MSC-2 cells at p5. Cell proliferation likewise arrested early, after 25 PDs. Early exhaustion of division potential is most likely connected to donor age: the other donors were 20–30 years old, whereas donor MSC-2 was 40 years of age. Similar findings correlating higher donor age with lower proliferative capacity have been reported by others [41, 42]. These results demonstrate that an increased cell size rather than PD number could indeed serve as a useful indicator of unanticipated senescence in MSC cultures.

A number of studies have correlated MSC morphology with proliferation or differentiation potential. Mets and Verdonk showed that a population of MSCs consisted of two types of cells, which could be separated by their size and proliferative capacity [12]. These findings were complemented by identification of very small and rapidly replicating MSCs, with divergent surface epitopes and high differentiation potential [5]. Automated microscopy and image analysis tools that resembled our setup have also been applied to MSC cultures in other studies [43–45]. Whitfield and colleagues [43] imaged MSCs continuously over 6 days and were able to form lineage maps arising from single cells to describe the onset of heterogeneity in MSC populations. They defined 7000 μm^2^ as a threshold area for large cells and found all such cells to be β-galactosidase positive. In our study, large cell sizes, 8744 ± 2494 μm^2^, were found among pSEN (passages 6–9) cells and the highest SA-β-gal activity was detected at passage 7 and thereafter.

The morphology of MSCs is highly predictive of their mineralization and immunosuppressive capacities [44, 45]. Small size, high colony-forming unit capacity and high proliferation rates were associated with a lower rate of telomere shortening in another study, which described predictive characteristics for MSC potency [46]. Measurement of cellular thickness using atomic force microscopy (AFM) can be used to analyze cell size to discover the relation of cell shape to the proliferative potential. Katsube and colleagues [47] found MSCs with high proliferative capacity to be thick, but became thin when the capacity was lost. Cells with low proliferative capacity were reported to be β-galactosidase positive and expressing elevated levels of the senescence-associated genes *p16*^*INK4a*^, *p21*^*Cip1/Waf1*^, and *MMP-1*. Although AFM is an ideal method for measuring cellular thickness, its usefulness is limited due to the technical complexity and its tendency to cause cell damage.

### Telomeres shorten at a constant rate during MSC culture

Mean telomere lengths were measured using TRF analysis. Starting from 8.2 ± 0.3 kbp at p1, the telomeres of cells collected at pSEN had been trimmed down to 6.8 ± 0.6 kbp in length, shortening at an approximately constant rate of 36.1 ± 12.4 bp at each cell division. No clear threshold value for telomere length regarding the onset of senescence could be observed. A more precise correlation of telomere shortening and senescence may require quantifying the length of the shortest instead of the average telomere length in the cells, which is known to trigger replicative senescence [17, 48]. However, our results confirm that telomere shortening occurs during long-term MSC expansion as part of the cellular aging process.

### Expression of aging-related markers correlates with the increase in cell size

Correlation analysis visualized by a heat map and PC analysis revealed the interdependence between changes in the cell area and the manifestation of senescence-related markers. The positive correlations between mean cell area and increasing expression of p16^INK4a^ and SA-β-gal as well as negative correlations between cell area, ceasing proliferative capacity, and decreasing telomere length confirmed that screening of enlargement of cell size does indeed indicate the expression of senescence-related markers. An initial increase and late decrease in the expression of p21^Cip1/Waf1^ combined with the expression of p16^INK4a^ is typical of cells with replicative senescence, and has also been reported by others [21, 22].

### Senescence assessment as a part of quality control

Senescence is, on the one hand, an essential tumor suppressive mechanism, but on the other hand detrimental to the cell itself and its surroundings. Researchers and regulators agree that assessment of senescence should be included in the quality control of cell therapy products [2, 26, 30, 32]. Quality control testing depends on the clinical application, but must include tests for purity, identity, and potency, for example. For MSCs this includes at least sterility tests, karyotype analysis, and tests to verify fulfilled ISCT minimal criteria [29] and functional properties such as immunosuppressive capacity [49]. The use of passage numbers to describe cellular age is questionable due to variable plating densities and harvesting practices, and therefore population doubling numbers are a more suitable measure for cellular age. However, our study suggests that MSC cultures grown under identical conditions vary in their proliferation potential and onset of the aging process. Thus, absolute PD limits to define good-quality MSCs are of limited value as quality criteria, and passage numbers are even less suitable. On the other hand, each cell product should be screened for its content of senescent cells to determine its quality.

Detection of senescence-associated DNA methylation changes provides an accurate assessment of the extent of senescence and can be used to evaluate the cell product, but the methodology is cumbersome [30, 50]. Phase-contrast microscopes qualified for documentation combined with supervised machine learning applications could, however, be a labor-saving and cost-effective option to screen MSC cultures [51, 52] once a prediction model has been established. From the regulators’ point of view, the use of senescence tests are not currently considered mandatory for the release of cell batches. However, they emphasize the need for manufacturers to assess the manifestations of senescence in their product [26].

### Future prospects in using imaging technology in cell manufacturing process

Methods to separate MSCs with different morphologies have concentrated on flow cytometry and imaging-based methods, which allow the exact quantification of the cell area and other morphological parameters. Imaging applications with machine learning analysis and prediction models have already been successfully applied in biological studies [42–45, 52] and may also be utilized in clinical-grade cell manufacturing [53, 54]. Our results show that imaging analysis is able to detect aging in MSC cultures; aging-related increase in cell area can be easily and reproducibly detected. A variety of methods are available for imaging applications, but most methods, such as ours, require sample collection, fixation, and staining steps which are difficult to integrate into the manufacturing process. Label-free imaging in combination with machine learning methods [53] could provide the ideal combination for integrating noninvasive real-time morphological image analysis into routine cell manufacturing.

## Conclusions

In this study, we quantified changes in cell morphology in long-term MSC cultures and found that cell area is the most significant and practical parameter to describe the morphological changes that accompany cell aging. We also showed that cell area correlates with the manifestation of senescence-associated markers. We conclude that image analysis is able to detect aging-related changes in cell morphology and can be applied in clinical-grade manufacturing and can thus provide a robust tool for MSC quality control.

## Additional files

Additional file 1: Table S1. Surface antigen expression of the MSCs. All cells (*n* = 6) expressed typical surface antigens of MSCs. In distinction to ISCT minimal criteria, MSCs expressed variable levels of HLA-DR antigen. (DOCX 13 kb)

Additional file 2: Figure S1. Osteogenic differentiation of the MSCs. MSCs from six donors differentiated into osteoblasts. A) 100% confluent undifferentiated control cells for osteogenic differentiation. B) Mineralization was detected by von Kossa staining in MSCs differentiated to osteoblasts. Images were acquired using 10× magnification. (PDF 162 kb)

Additional file 3: Figure S2. Adipogenic differentiation of the MSCs. MSCs from six donors differentiated into adipocytes. A) 100% confluent undifferentiated control cells for adipogenic differentiation. B) Adipogenic differentiation was detected by Sudan III staining. Images were acquired using 4× and 10× magnifications. (PDF 160 kb)

Additional file 4: Figure S3. Mean cell area in small-scale and large-scale long-term cultures. Long-term cultures of MSCs were performed in small-scale in culture flasks (*n* = 3) and in large-scale (*n* = 3) in two-layer cell stacks. Bonferroni-corrected Student’s *t* test, **p < 0.01; ANOVA, *p < 0.05; *ns* not significant. (PDF 33 kb)

Additional file 5: Figure S4. Western blot analysis of aging-related expression of p16^INK4a^ and p21^Cip1/Waf1^. Western blot analysis was performed from cell lysate samples containing 20 μg total protein for six donors. β-actin was used as a loading control. (PDF 59 kb)

Additional file 6: Figure S5. Telomere length measurement using TRF analysis. A representative figure of the measurement of the mean telomere length. The measurement was performed for MSC-1 to MSC-6 (*n* = 6). Each sample was analyzed in triplicate and mean telomere length was calculated according to [39]. (PDF 40 kb)

## Abbreviations

AFM: Atomic force microscopy
ANOVA: Analysis of variance
DIG: Digoxigenin
GVHD: Graft-versus-host disease
ISCT: International Society for Cellular Therapy
MSC: Mesenchymal stromal cell
OD: Optical density
p: Passage
PBS: Phosphate-buffered saline
PC: Principal component
PD: Population doubling
pSEN: Senescent passages (6–9)
SASP: Senescence-associated secretory phenotype
SA-β-gal: Senescence-associated β-galactosidase
TRF: Terminal restriction fragment
