# Supplementary material for: Automated image analysis detects aging in clinical-grade mesenchymal stromal cell cultures
Source: Stem Cell Res Ther. 2018 Jan 10;9:6. doi: 10.1186/s13287-017-0740-x (PMC5763576; doi:10.1186/s13287-017-0740-x)
Supplement: Supplementary file 1 — Surface antigen expression of the MSCs. All cells (n = 6) expressed typical surface antigens of MSCs. In distinction to ISCT minimal criteria, MSCs expressed variable levels of HLA-DR antigen. (DOCX 13 kb) [file 13287_2017_740_MOESM1_ESM.docx]

**Table S1.**

| **Antigen (%)** | **MSC-1** | **MSC-2** | **MSC-3** | **MSC-4** | **MSC-5** | **MSC-6** |
| --- | --- | --- | --- | --- | --- | --- |
| **CD13** | 99.9 | 100 | 98.6 | 99.6 | 99.9 | 99.9 |
| **CD29** | 99.7 | 99.9 | 99.9 | 100 | 100 | 100 |
| **CD44** | 99.8 | 99.9 | 98.5 | 99.5 | 99.9 | 99.9 |
| **CD49e** | 99.9 | 100 | 98.6 | 98.8 | 99.8 | 99.9 |
| **CD73** | 99.9 | 100 | 99.9 | 99.8 | 99.9 | 100 |
| **CD90** | 99.9 | 99.7 | 99.9 | 99.9 | 100 | 100 |
| **CD105** | 99.8 | 99.5 | 97.9 | 99.9 | 99.8 | 99.8 |
| **HLA-ABC** | 99.9 | 98.6 | 98.9 | 99.1 | 97.1 | 99.8 |
| **HLA-DR** | 47.4 | 7.5 | 18.5 | 15.6 | 3.4 | 47.8 |
| **CD14** | <1 | <1 | <1 | <1 | <1 | <1 |
| **CD19** | <1 | <1 | <1 | <1 | <1 | <1 |
| **CD34** | <1 | <1 | <1 | <1 | <1 | <1 |
| **CD45** | <1 | <1 | <1 | <1 | <1 | <1 |
