# Supplementary figures and images for: Automated image analysis detects aging in clinical-grade mesenchymal stromal cell cultures
Source: Stem Cell Res Ther. 2018 Jan 10;9:6. doi: 10.1186/s13287-017-0740-x (PMC5763576; doi:10.1186/s13287-017-0740-x)

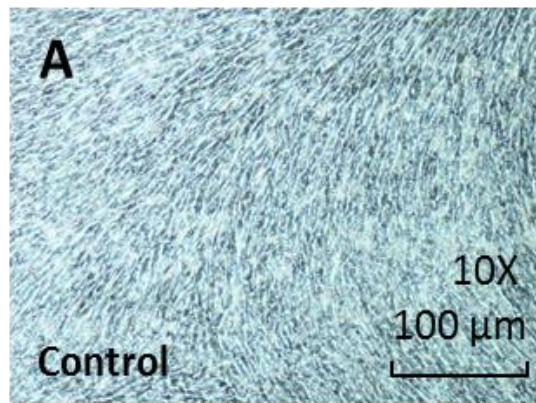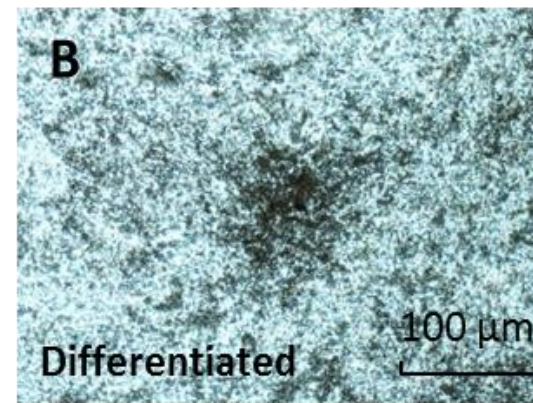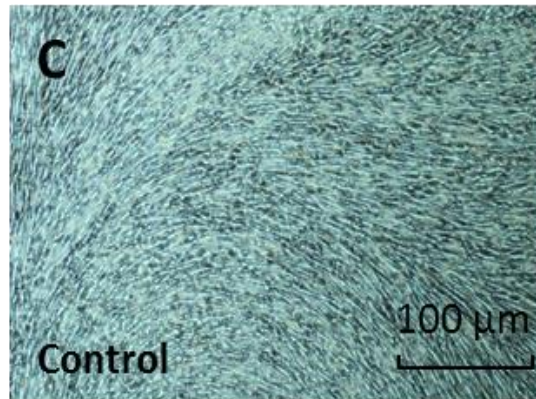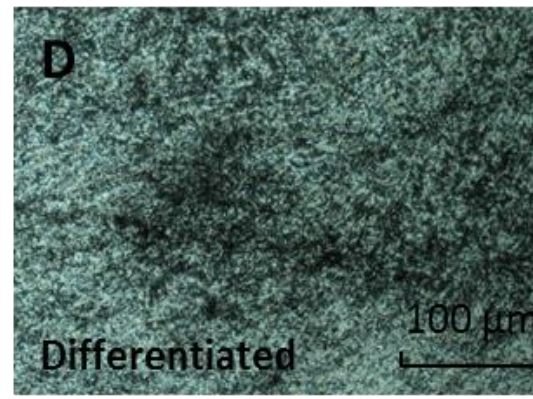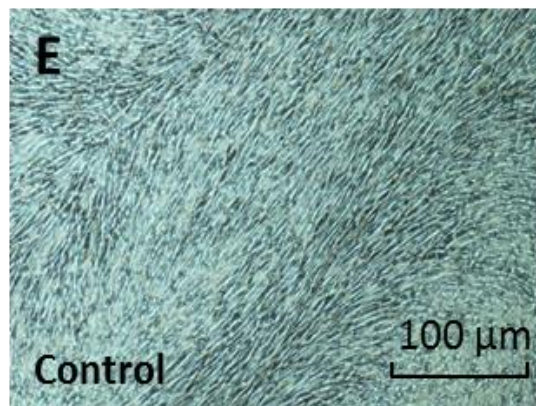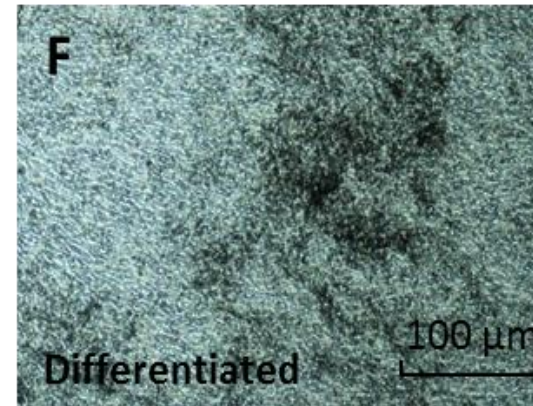

**Supplementary Figure 1. Oja S. *et al.***

Supplement: Supplementary file 2 — Osteogenic differentiation of the MSCs. MSCs from six donors differentiated into osteoblasts. A) 100% confluent undifferentiated control cells for osteogenic differentiation. B) Mineralization was detected by von Kossa staining in MSCs differentiated to osteoblasts. Images were acquired using 10× magnification. (PDF 162 kb) [file 13287_2017_740_MOESM2_ESM.pdf]

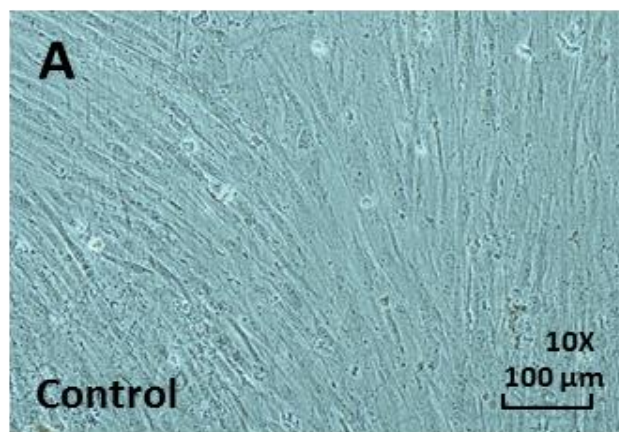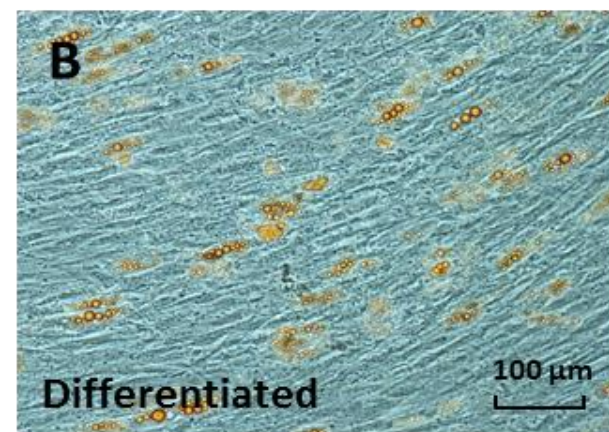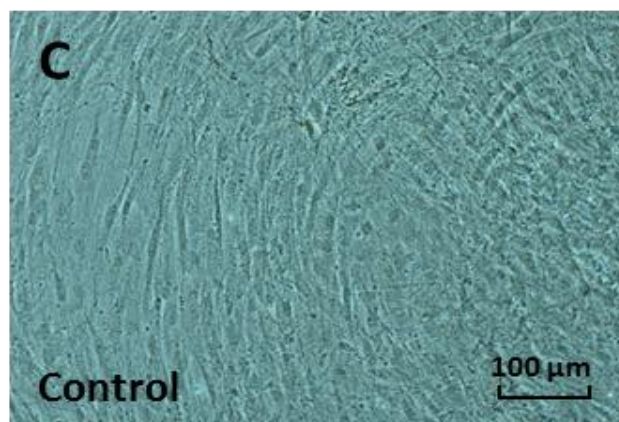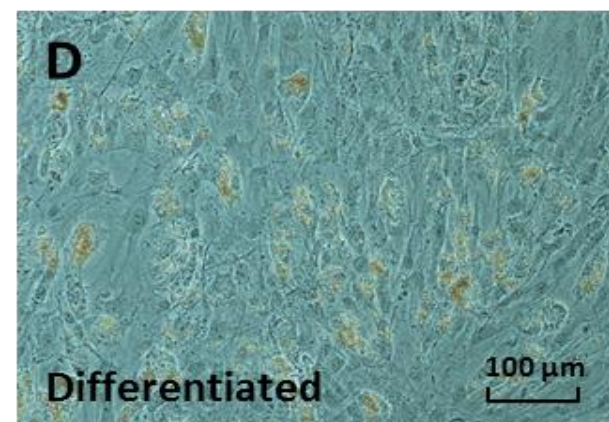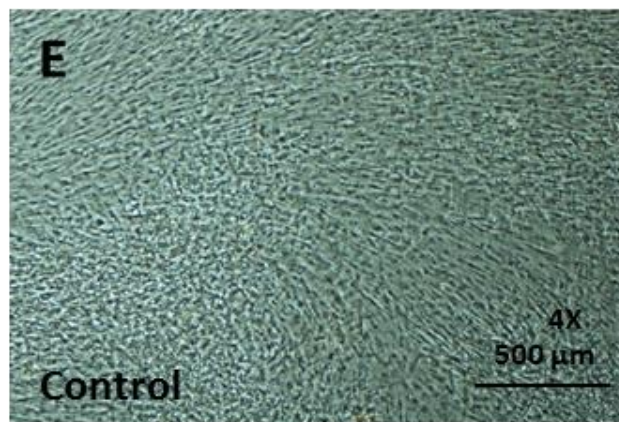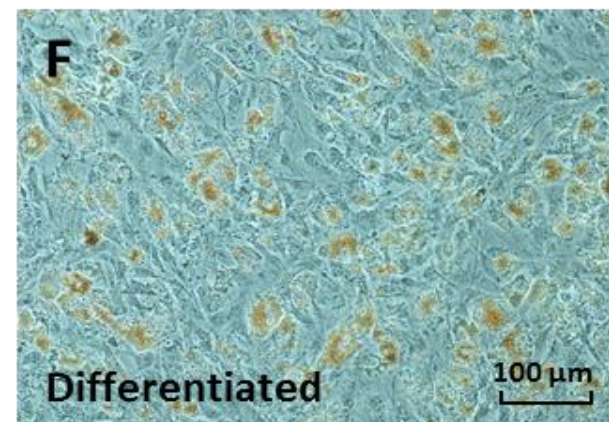

Supplementary Figure 2. Oja S. *et al.*

Supplement: Supplementary file 3 — Adipogenic differentiation of the MSCs. MSCs from six donors differentiated into adipocytes. A) 100% confluent undifferentiated control cells for adipogenic differentiation. B) Adipogenic differentiation was detected by Sudan III staining. Images were acquired using 4× and 10× magnifications. (PDF 160 kb) [file 13287_2017_740_MOESM3_ESM.pdf]

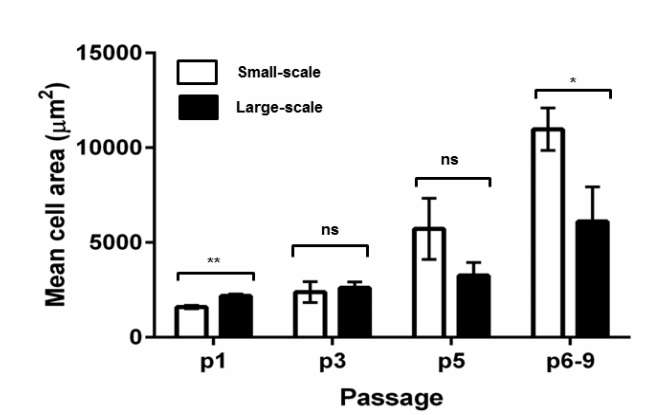

**Supplementary Figure 3. Oja S. *et al.***

Supplement: Supplementary file 4 — Mean cell area in small-scale and large-scale long-term cultures. Long-term cultures of MSCs were performed in small-scale in culture flasks (n = 3) and in large-scale (n = 3) in two-layer cell stacks. Bonferroni-corrected Student’s t test, **p < 0.01; ANOVA, *p < 0.05; ns not significant. (PDF 33 kb) [file 13287_2017_740_MOESM4_ESM.pdf]

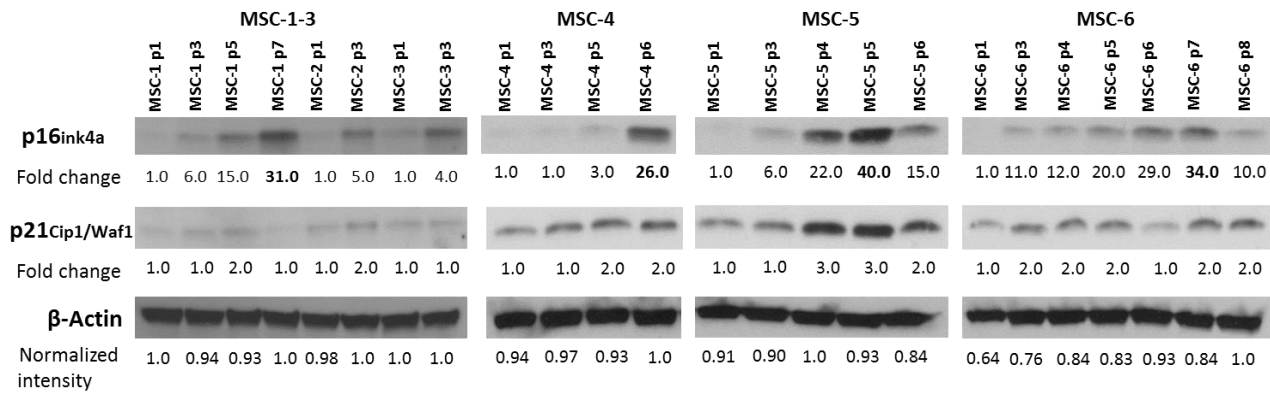

**Supplementary Figure 4. Oja S. *et al.***

Supplement: Supplementary file 5 — Western blot analysis of aging-related expression of p16INK4a and p21Cip1/Waf1. Western blot analysis was performed from cell lysate samples containing 20 μg total protein for six donors. β-actin was used as a loading control. (PDF 59 kb) [file 13287_2017_740_MOESM5_ESM.pdf]

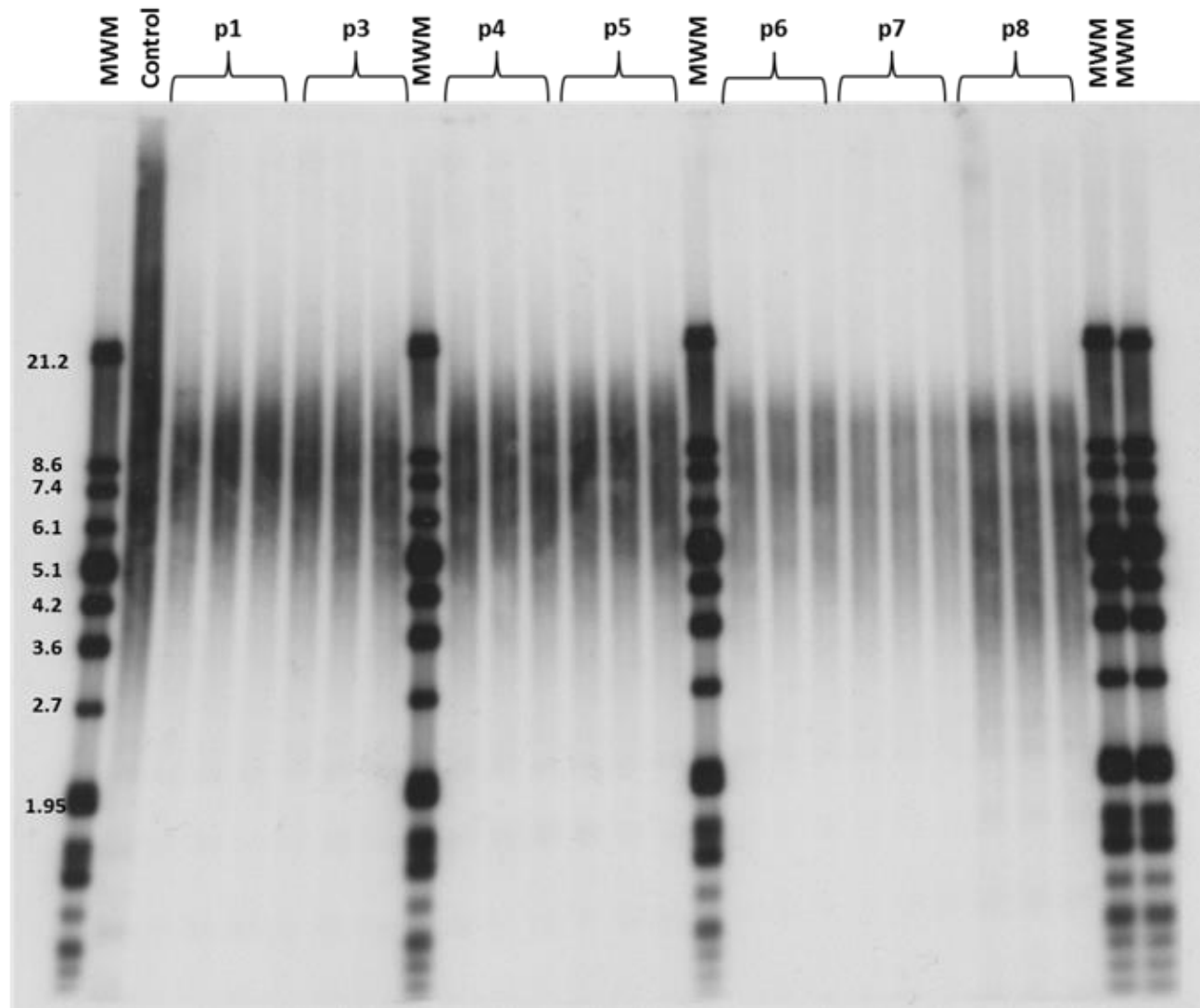

Supplementary Figure 5. Oja S. *et al.*

Supplement: Supplementary file 6 — Telomere length measurement using TRF analysis. A representative figure of the measurement of the mean telomere length. The measurement was performed for MSC-1 to MSC-6 (n = 6). Each sample was analyzed in triplicate and mean telomere length was calculated according to [39]. (PDF 40 kb) [file 13287_2017_740_MOESM6_ESM.pdf]
